# Incidence of Adenomyosis in Hysterectomy Specimens Performed for Benign Uterine Pathologies: A Six-Year Retrospective Analysis from a Tertiary Center in Romania

**DOI:** 10.3390/diagnostics16213448

**Published:** 2026-10-22

**Authors:** Andreea Ioana Pruteanu, Ioana Păvăleanu, Ana Maria Haliciu, Ana Maria Mihoci, Raluca Anca Balan, Emil Anton, Sorana Caterina Anton, Demetra Gabriela Socolov, Razvan Vladimir Socolov

**Affiliations:** 1 “Grigore T. Popa” University of Medicine and Pharmacy, 16 University Street, 700115 Iasi, Romania; andreea.ioana.dragu@gmail.com (A.I.P.); emil.anton@yahoo.com (E.A.); sorana.anton@yahoo.com (S.C.A.); socolov.razvan@gmail.com (R.V.S.); 2 “Elena Doamna” Clinical Hospital of Obstetrics and Gynecology, 49 Elena Doamna Street, 700398 Iasi, Romania; 3 Department of Mother and Child Medicine, “Grigore T. Popa” University of Medicine and Pharmacy, 16 University Street, 700115 Iasi, Romania; 4 Department of Morpho-Functional Sciences I, “Grigore T. Popa” University of Medicine and Pharmacy, 16 University Street, 700115 Iasi, Romania; 5 Clinical Hospital of Obstetrics and Gynecology “Cuza Vodă”, 700038 Iasi, Romania

**Keywords:** adenomyosis, hysterectomy, prevalence, preoperative diagnosis, MUSA criteria, transvaginal ultrasound, benign uterine pathology, endometrial hyperplasia

## Abstract

**Background/Objectives:** Adenomyosis is a common yet frequently underdiagnosed cause of gynecological morbidity, and its definitive diagnosis still relies on histopathological examination of hysterectomy specimens. We aimed to determine the histopathological prevalence of adenomyosis in hysterectomy specimens removed for benign indications, characterize preoperative referral diagnoses and concurrent histopathological findings, and compare our results with the contemporary literature. **Methods:** We retrospectively analyzed 430 consecutive abdominal hysterectomies performed for benign gynecological indications at a tertiary referral center in Iasi, Romania, between January 2019 and October 2024. Adenomyosis was defined histopathologically. An “incidental” finding was defined as the absence of adenomyosis in the preoperative referral diagnosis, without re-review of the original ultrasound reports. We report prevalence and the incidental rate with Wilson-score 95% confidence intervals (CIs); we used the chi-square test with Yates continuity correction for categorical comparisons and calculated odds ratios (ORs), with correction for multiple comparisons. **Results:** Adenomyosis was identified in 188 of 430 specimens (43.7%; 95% CI 39.1–48.4%), of which 186 (98.9%; 95% CI 96.2–99.7%) were incidental; adenomyosis was recorded in the referral diagnosis in only 2 cases (1.1%). Endometrial hyperplasia was the only co-finding significantly associated with adenomyosis (OR 2.30; 95% CI 1.48–3.55; *p* = 0.0002), an unadjusted univariate association that remained significant after multiple-comparison adjustment (adjusted *p* = 0.0014). No referral diagnosis discriminated adenomyosis status; leiomyoma was frequent in both groups (75.5% vs. 69.0%; not significant). Mean age was 50.0 ± 7.6 years, with no between-group difference (*p* = 0.112). **Conclusions:** Approximately two in five women undergoing hysterectomy for benign disease have adenomyosis, and it is rarely suspected preoperatively. Because this study did not test whether structured sonographic assessment improves detection, prospective evaluation of the revised 2022 Morphological Uterus Sonographic Assessment (MUSA) criteria with imaging–histopathology correlation is warranted.

## 1. Introduction

Adenomyosis is a benign uterine disorder characterized by the presence of endometrial glands and stroma within the myometrium. In the literature, diagnostic definitions commonly require that ectopic endometrial tissue lie at least 2.5 mm below the endometrial–myometrial junction or involve more than one third of the myometrial thickness to distinguish true myometrial invasion from superficial extension of the basalis layer [1,2]. These thresholds are not universally applied in routine diagnostic practice, an issue directly relevant to the present series and addressed in Section 2.4. The definition nevertheless emphasizes the invasive nature of the disease, and histopathology remains the reference standard for definitive diagnosis.

Adenomyosis is a significant source of morbidity in women of reproductive age. It is a common cause of abnormal uterine bleeding (AUB), often heavy, prolonged, and poorly responsive to medical therapy, and formally recognized in the PALM-COEIN classification. Uterine enlargement may cause bulk symptoms such as pelvic pressure, urinary frequency, and constipation, while severe dysmenorrhea and chronic pelvic pain can substantially impair quality of life. Adenomyosis has also been associated with impaired fertility and adverse obstetric outcomes, including miscarriage, preterm birth, and fetal growth restriction, through mechanisms involving altered uterine contractility, impaired implantation, and chronic inflammation [3,4]. Because these symptoms overlap with those of leiomyomas and endometriosis, diagnosis and management are often complicated.

Despite its prevalence and clinical importance, adenomyosis remains substantially underdiagnosed before surgery. For many years, the condition was identified almost exclusively through retrospective histopathological analysis of hysterectomy specimens, with reported prevalence ranging from 20% to more than 50% among women undergoing hysterectomy for benign indications [5,6]. Recent cohort studies and meta-analyses confirm that this high prevalence persists: adenomyosis is documented in approximately 40–50% of hysterectomies performed for AUB or symptomatic leiomyomas, yet preoperative clinical or imaging suspicion is recorded in only a minority of cases [5,6,7,8]. This persistent diagnostic gap means that many women undergo definitive surgery without a clear preoperative diagnosis, limiting opportunities for individualized counseling, fertility preservation, and uterus-preserving alternatives.

Transvaginal ultrasound (TVUS) is the recommended first-line imaging modality for suspected adenomyosis because of its wide availability and favorable cost-effectiveness. However, its diagnostic performance is highly operator-dependent. Recognizing the characteristic features—myometrial cysts, hyperechoic islands, subendometrial echogenic lines and buds, a globular uterine configuration, and junctional-zone irregularity—requires considerable expertise and is influenced by equipment quality, scanning technique, and familiarity with standardized descriptors. The Morphological Uterus Sonographic Assessment (MUSA) consensus, first published in 2015 and revised in 2022, sought to improve reproducibility by classifying features as direct or indirect and proposing diagnostic thresholds [9]. Nevertheless, validation studies report only moderate inter-observer agreement and variable sensitivity, particularly when leiomyomas coexist, and show a clear gradient in performance according to examiner experience [10,11]. In routine practice, ultrasound reports often omit a systematic description of these features, especially when a more conspicuous abnormality already accounts for the patient’s symptoms, which contributes to missed or delayed diagnoses. Experienced sonologists are considerably more likely to identify adenomyosis than general practitioners of ultrasound, underscoring the influence of operator expertise [12].

This retrospective study aimed to determine the histopathological prevalence of adenomyosis in a consecutive series of hysterectomy specimens collected over six years (2019–2024) at a tertiary gynecological center in Iasi, Romania. In addition to the overall prevalence and demographic profile, we sought to characterize preoperative referral diagnoses, identify concurrent histopathological findings, and quantify the proportion of cases in which adenomyosis was an incidental finding. We interpreted the findings by comparing them directly with published international evidence.

## 2. Materials and Methods

### 2.1. Study Design and Setting

This retrospective cohort study was conducted at the “Elena Doamna” Clinical Hospital of Obstetrics and Gynecology, a tertiary referral center in Iasi, Romania. It included all consecutive abdominal hysterectomies performed for benign gynecological indications from January 2019 to October 2024, identified through the hospital’s surgical registry and linked to electronic medical records. The study was conducted in accordance with the Declaration of Helsinki [13] and approved by the Ethics Committee of the “Elena Doamna” Clinical Hospital of Obstetrics and Gynecology, Iasi, Romania (protocol code 10940, approval date 30 December 2024). Because the analysis used retrospective, fully de-identified data, individual informed consent was waived. The study is reported in accordance with the STROBE statement for observational research.

### 2.2. Study Population and Inclusion Criteria

All adult female patients (aged ≥18 years) who underwent abdominal hysterectomy for benign indications were eligible for inclusion. Procedures were identified using the Romanian national operative procedure codes M02904 (total hysterectomy with bilateral adnexectomy), M02901 (total hysterectomy), and M02902 (total hysterectomy with unilateral adnexectomy). Only cases with a complete histopathological report of the hysterectomy specimen were included. Patients were excluded if the hysterectomy was performed for a gynecological malignancy known or suspected preoperatively, if histopathological data were missing, or if medical records were insufficient for reliable data extraction. Eligibility was therefore determined by the indication for surgery as documented before the operation, not by the final histopathological result. Consequently, specimens in which a malignancy was recognized only on final histopathology (occult malignancy) remained in the cohort; the handling of these cases is detailed in Section 3.4.

### 2.3. Data Collection

Data were extracted from the institutional hysterectomy registry and the corresponding electronic medical records. Collected variables included patient age at surgery, preoperative referral diagnosis, operative procedure code, surgeon identifier, procedure date, and the complete histopathological report. The preoperative referral diagnosis served as a proxy for the clinical indication for surgery and was used to determine whether adenomyosis had been suspected before the operation. All histopathological reports were reviewed for adenomyosis and for any concurrent uterine or adnexal findings.

### 2.4. Histopathological Assessment and Diagnostic Criteria

All hysterectomy specimens were processed in the host institution’s pathology department according to routine diagnostic practice in effect during the study period. Specimens were examined macroscopically, and myometrial sections were taken at the reporting pathologist’s discretion, guided by the gross appearance of the uterus. No fixed, protocol-mandated number of myometrial blocks was required, so the number of sections examined per uterus varied across specimens.

Adenomyosis was recorded when the reporting pathologist identified endometrial glands and stroma within the myometrium. We wish to state explicitly that no single quantitative depth threshold—for example, a minimum distance of 2.5 mm below the endometrial–myometrial junction, a depth exceeding one low-power field, or involvement of more than one third of the myometrial wall—was prospectively mandated or uniformly recorded in the reports. The diagnosis was rendered descriptively, consistent with routine reporting practice. The morphological thresholds cited in the Introduction therefore represent definitions proposed in the literature rather than criteria that can be verified as having been uniformly applied in this series. Reports were issued by a single attending pathologist, working with the hospital’s resident team, over the six-year study period, and no centralized retrospective slide review was undertaken for this study. The consequences of both the absence of a mandated threshold and the non-protocolized sampling for the prevalence estimate are considered in Section 4.9.

### 2.5. Variable Definitions

The primary outcome was the overall histopathological prevalence of adenomyosis. Secondary outcomes included the rate of preoperative identification of adenomyosis, the frequency of histopathological co-findings, and comparisons between cases with and without adenomyosis. An “incidental” finding was defined as histopathologically confirmed adenomyosis in the absence of any mention of adenomyosis in the preoperative referral diagnosis. Because the original ultrasound reports were not re-reviewed, this definition reflects the absence of documented preoperative clinical suspicion rather than the failure of any specific imaging modality. Referral diagnoses were classified by keyword extraction into the following categories: uterine fibroid/fibromatosis, abnormal uterine bleeding, genital prolapse, ovarian cyst, cervical dysplasia/cervicitis, pelvic or abdominal tumor, and postmenopausal bleeding. Histopathological co-findings were grouped as leiomyoma, endometrial hyperplasia (with subtype where specified), endometrial polyp, chronic endometritis, ovarian cysts or other adnexal pathology, endometriosis, and malignancy.

### 2.6. Statistical Analysis

All analyses were performed in Python 3.12 (Python Software Foundation, Wilmington, DE, USA) with SciPy 1.11 (open-source scientific library; https://scipy.org). Normality of the age distribution was assessed using scipy.stats.shapiro. Because age was non-normally distributed in both the adenomyosis group (W = 0.906, *p* < 0.001) and the non-adenomyosis group (W = 0.946, *p* < 0.001), the Mann–Whitney U test (scipy.stats.mannwhitneyu, alternative = ‘two-sided’, default asymptotic method with continuity correction) was pre-specified as the primary test for the between-group comparison of age. An independent two-sample Student’s *t*-test (scipy.stats.ttest_ind) was computed in addition, solely as a confirmatory sensitivity analysis, and is reported as such throughout. Categorical comparisons were performed with scipy.stats.chi2_contingency applied to 2 × 2 contingency tables, with the Yates continuity correction retained at its default setting (correction = True); the χ^2^ statistics reported in Section 3.4 are therefore continuity-corrected. Odds ratios (ORs) and their 95% confidence intervals (CIs) were derived from the same 2 × 2 tables using the Woolf logit method. Proportions are reported with 95% CIs calculated by the Wilson score method without continuity correction. Continuous data are presented as mean ± standard deviation (SD) and as median with interquartile range (IQR). All tests were two-sided, and α was set at 0.05.

Because seven histopathological co-findings were compared between groups, the corresponding *p*-values were corrected for multiplicity using the Benjamini–Hochberg false-discovery-rate procedure, with the Bonferroni and Holm–Bonferroni procedures applied as more conservative sensitivity analyses; both unadjusted and adjusted *p*-values are reported in Section 3.4. The comparisons of referral-diagnosis categories (Section 3.3) were exploratory and are reported unadjusted, since none reached nominal significance and correction would not alter the conclusion.

Age was missing for 12 of the 430 patients (2.8%). These cases were excluded only from age comparisons (complete-case analysis) and retained in all other analyses. Following the inclusion criteria, a preoperative referral diagnosis and a complete histopathological report were available for all 430 included patients, so no other variable had missing values. No a priori sample-size calculation was performed; all consecutive eligible cases in the study period were included, which fixed the attainable precision of the prevalence estimate. No generative artificial intelligence tools were used in the study design, data collection, analysis, or interpretation.

## 3. Results

### 3.1. Study Population and Overall Prevalence

Over the six-year period from 2019 to 2024, 430 hysterectomy cases meeting the inclusion criteria were identified. The most common procedure was total hysterectomy with bilateral adnexectomy (M02904; *n* = 280, 65.1%), followed by total hysterectomy (M02901; *n* = 100, 23.3%) and total hysterectomy with unilateral adnexectomy (M02902; *n* = 50, 11.6%). These procedures were performed by 26 surgeons.

Adenomyosis was identified histopathologically in 188 of 430 cases, yielding a prevalence of 43.7% (95% CI 39.1–48.4%). Of these, only 2 cases (1.1%) had adenomyosis recorded in the preoperative referral diagnosis, whereas the remaining 186 (98.9%; 95% CI 96.2–99.7%) were incidental histopathological findings. Demographic and procedural characteristics are summarized in Table 1.

### 3.2. Age Distribution

The mean age of the entire cohort was 49.7 ± 8.6 years (median 48 years, IQR 44–53, range 24–77). In the adenomyosis group, the mean age was 50.0 ± 7.6 years (median 49 years, IQR 46–51, range 32–77), compared with 49.4 ± 9.4 years (median 47 years, IQR 43–53, range 24–77) in the non-adenomyosis group. This difference was not statistically significant in the primary analysis (Mann–Whitney U = 23,474, *p* = 0.112), a result confirmed by the sensitivity *t*-test (t = 0.741, *p* = 0.459). The 40–49-year age group was the most represented among adenomyosis cases (107/188, 56.9%), followed by the 50–59-year group (41/188, 21.8%). No adenomyosis cases were identified in patients younger than 30 years.

### 3.3. Preoperative Referral Diagnoses

Table 2 presents the distribution of preoperative referral diagnoses. In the adenomyosis group, the most common referral indication was uterine fibroid or fibromatosis (127/188, 67.6%), followed by abnormal uterine bleeding (104/188, 55.3%), genital prolapse (18/188, 9.6%), cervical dysplasia or cervicitis (16/188, 8.5%), and ovarian cyst (15/188, 8.0%). No referral diagnosis category differed significantly between the adenomyosis and non-adenomyosis groups (all *p* ≥ 0.110). Critically, only 2 of 188 adenomyosis cases (1.1%) had adenomyosis explicitly stated in the preoperative referral diagnosis, underscoring the overwhelmingly incidental nature of the findings.

### 3.4. Histopathological Co-Findings

Histopathological co-findings are summarized in Table 3. Leiomyoma was the most common co-pathology in both the adenomyosis group (142/188, 75.5%) and the non-adenomyosis group (167/242, 69.0%), with no significant between-group difference (OR 1.39, 95% CI 0.90–2.13; *p* = 0.166). Endometrial hyperplasia was identified in 67 adenomyosis cases (35.6%) compared with 47 non-adenomyosis cases (19.4%), and was the only statistically significant association (OR 2.30, 95% CI 1.48–3.55; χ^2^ = 13.461, *p* = 0.0002). Among the 67 hyperplasia cases in the adenomyosis group, 43 were simple without atypia, 2 showed atypia, and 22 were of unspecified subtype. Ovarian cysts or another ovarian pathology were present in 74 adenomyosis cases (39.4%) and 87 non-adenomyosis cases (36.0%), with no significant difference (*p* = 0.532). Endometrial polyp, endometritis, endometriosis, and malignancy showed no significant between-group differences.

After correcting for multiple comparisons across the seven co-findings, the association with endometrial hyperplasia remained significant (Benjamini–Hochberg-adjusted *p* = 0.0014; Bonferroni- and Holm-adjusted *p* = 0.0014), whereas no other comparison reached significance (all adjusted *p* ≥ 0.58).

Malignancy was identified in 11 adenomyosis cases (5.9%), comprising 9 carcinomas and 2 sarcomas (including 1 leiomyosarcoma), compared with 19 non-adenomyosis cases (7.9%; *p* = 0.537). All 30 malignancies were occult: in each case, the hysterectomy had been performed for a documented benign indication, and the malignancy was recognized only on final histopathological examination of the specimen. No patients with malignancies known or suspected before surgery were included. These cases were deliberately retained in the analysis because excluding them would have removed patients based on an outcome that could not have been known at the time of the decision to operate, thereby biasing the cohort.

### 3.5. Summary of Key Findings

In this six-year series, adenomyosis was histopathologically identified in 43.7% of hysterectomy specimens, and 98.9% of these cases were incidental findings that had not been suspected preoperatively. The only significant association was with endometrial hyperplasia (OR 2.30; *p* = 0.0002; adjusted *p* = 0.0014). No referral diagnosis category significantly distinguished cases with adenomyosis from those without, and age did not differ meaningfully between the groups. These results indicate that adenomyosis should be anticipated across the full spectrum of hysterectomy indications and patient ages.

## 4. Discussion

### 4.1. Prevalence in the Context of the Literature

The histopathological prevalence of adenomyosis in our cohort of 430 cases was 43.7% (95% CI 39.1–48.4%), which falls within the internationally reported range and is closely comparable to contemporary series. Krentel and De Wilde reported adenomyosis in 42.0% of 307 hysterectomy specimens removed for benign conditions, with the highest rates among women operated on for bleeding disorders and dysmenorrhea [5]. Lonky et al. found a prevalence of 45.9% among 370 women undergoing hysterectomy for AUB associated with leiomyomas, irrespective of the presence of submucous fibroids [6], and Abed et al. documented a prevalence of 52% in a series from Sana’a, Yemen [8]. Wang et al.’s systematic review and meta-analysis estimated a pooled prevalence of 42.3% (95% CI 35.8–48.9%) among women undergoing hysterectomy for benign conditions, compared with 31.9% for malignant indications, and reported that histopathology (35.1%) and magnetic resonance imaging (MRI, 35.0%) detect adenomyosis more frequently than ultrasound (30.7%) [7]. Our figure of 43.7% therefore sits squarely within this contemporary range, supporting the view that adenomyosis affects approximately two in five women undergoing hysterectomy for benign disease.

The most striking finding is that 98.9% of adenomyosis cases (186/188) were diagnosed solely by histopathology and were not suspected preoperatively. It should be emphasized that, in the present study, “incidental” status was defined by the absence of adenomyosis in the preoperative referral diagnosis rather than by a structured re-review of the original ultrasound reports; our data therefore reflect the absence of documented preoperative clinical suspicion and cannot, on their own, quantify the sensitivity of TVUS. This near-complete absence of preoperative suspicion is nonetheless consistent with the limited ultrasound performance reported in the literature, including the lower detection rate of ultrasound relative to histopathology and MRI in the meta-analysis by Wang et al. [7] and the insensitivity of two-dimensional ultrasound in fibroid-affected uteri described by Lonky et al. [6]. The annual prevalence in our series ranged from 30.9% to 62.2%, a variation likely reflecting differences in case mix and pathological processing rather than true temporal trends.

### 4.2. Age Profile in Relation to the Literature

The mean age of adenomyosis cases was 50.0 ± 7.6 years, with the 40–49-year group predominating (56.9%), consistent with the well-established perimenopausal peak [1]. Age did not differ markedly between the adenomyosis and non-adenomyosis groups (*p* = 0.112 in the primary analysis), indicating that adenomyosis should be considered across the full age spectrum of hysterectomy patients, including postmenopausal women. Our cohort is notably older than that of Lonky et al. [6], likely because their series was restricted to premenopausal women with fibroid-associated AUB, whereas ours comprises a broader, unselected surgical population.

### 4.3. Referral Diagnoses: Adenomyosis as a Silent Co-Pathology

No preoperative referral diagnosis category was significantly more common in adenomyosis than in non-adenomyosis cases. Uterine fibroid/fibromatosis remained the most frequent referral in both groups (67.6% vs. 64.5%, *p* = 0.570), as did abnormal uterine bleeding (55.3% vs. 51.7%, *p* = 0.510). This lack of discriminating power reflects the clinical reality that adenomyosis is a silent co-pathology in the vast majority of cases; its presence cannot be inferred from the referral diagnosis alone. These results reinforce the need for systematic preoperative imaging strategies to detect adenomyosis regardless of the primary indication.

### 4.4. Endometrial Hyperplasia: The Single Significant Co-Finding

Endometrial hyperplasia was significantly more prevalent in the adenomyosis group (35.6% vs. 19.4%; OR 2.30, 95% CI 1.48–3.55; *p* = 0.0002) and was the only statistically significant co-finding (Table 3). The association persisted after correction for multiple comparisons (adjusted *p* = 0.0014 by the Benjamini–Hochberg, Bonferroni and Holm procedures alike), indicating that it is unlikely to be a chance finding arising from the number of comparisons performed. This remains an unadjusted, univariate association: age, body mass index, parity and unopposed-estrogen exposure—all plausible confounders for both conditions—were not available in the dataset and could not be controlled for. No inference of an independent or causal relationship is therefore intended or warranted. The association is biologically plausible and would be compatible with a partly shared estrogen-dependent pathogenesis, since adenomyosis is characterized by elevated local estrogen production, progesterone resistance and persistent endometrial proliferative signaling [1]. The hypercoagulable, estrogenic–inflammatory milieu described in adenomyosis-associated heavy menstrual bleeding, with elevated plasma fibrinogen and D-dimer levels, may plausibly accompany such concurrent endometrial overgrowth [14]. A multivariable logistic regression adjusting for these confounders would be required to determine whether the association is independent.

The predominance of simple hyperplasia without atypia (43/67 cases, 64.2%) suggests a benign rather than a premalignant proliferative process. Nevertheless, atypia was identified in 2 cases (3.0%), and the overall co-occurrence rate of 35.6% supports heightened endometrial surveillance in women with known adenomyosis, particularly when heavy menstrual bleeding is the presenting symptom. This association has been infrequently reported in comparable published series, including that of Lonky et al. [6], and warrants confirmation in adequately adjusted cohorts.

### 4.5. Leiomyoma: High Co-Occurrence Without Discriminating Value

Leiomyoma was present in 75.5% of adenomyosis cases and in 69.0% of non-adenomyosis cases, with no statistically significant between-group difference (OR 1.39, *p* = 0.166; Table 3). Although the high co-occurrence is consistent with the expectation that these two conditions frequently coexist [1,6], the lack of statistical significance indicates that leiomyoma co-occurrence, while very common in adenomyosis, is also highly prevalent in uteri without adenomyosis and therefore cannot serve as a discriminating clinical marker. This point is central to the diagnostic problem discussed below. A comparison of all co-findings with the published literature is provided in Table 4.

### 4.6. Low Preoperative Suspicion and the Likely Under-Reporting of MUSA Criteria

The 98.9% incidental rate indicates that adenomyosis was rarely suspected before surgery. Because this rate was derived from the referral diagnosis rather than from an audit of the primary ultrasound reports, it should be interpreted as reflecting low documented clinical suspicion rather than as a direct measure of ultrasound sensitivity. That said, low preoperative suspicion is expected given the well-documented, operator-dependent performance of TVUS, whose sensitivity falls markedly in the presence of leiomyomas [6,9]. We further hypothesize that, in routine practice, the MUSA criteria for adenomyosis are often not documented when a more conspicuous pathology—most often a uterine fibroid—already accounts for the patient’s clinical presentation: once an obvious structural cause of bleeding or pelvic pain is identified, the examination is often concluded without a systematic search for, or reporting of, the direct and indirect MUSA features of adenomyosis. This interpretation is consistent with our data—uterine fibroid/fibromatosis was the dominant referral in both groups yet did not discriminate adenomyosis status, and preoperative documentation of adenomyosis was almost absent (1.1%)—but it remains an inference, because the present study did not re-review the original ultrasound examinations. The reported diagnostic performance of the available imaging modalities, as summarized in the literature, is presented alongside our referral-based detection rate in Table 5.

The 2022 revised MUSA criteria provide a validated framework that could narrow this gap. In external validation studies, Yavuz et al. reported that a cumulative MUSA feature score greater than 4 yielded the best prediction, with a sensitivity of 27.5%, a specificity of 90%, and an overall accuracy of 77% [10]. Biasioli et al. observed high specificity but low sensitivity for individual direct features, with specificities of 86–98% for myometrial cysts, 83–95.5% for hyperechoic islands, and 78% for hyperechoic lines, against corresponding sensitivities of only 47–55%, 12–54%, and 51% [15]. Maghsoudlou et al., in a blinded single-expert validation against histopathology, showed that the composite of direct MUSA findings was 100% sensitive for adenomyosis [11]. These performance figures are from the cited studies, not from our cohort. Reproducibility remains a limitation: Kadam et al. found only modest intra- and inter-observer reproducibility in identifying at least one direct feature and recommended further refinement of the definitions [16].

### 4.7. Divergence in Reported MUSA Diagnostic Performance

The performance estimates summarized above are not directly comparable, and the apparent contradiction between them—a composite of direct features reported as 100% sensitive [11], versus sensitivities as low as 12–55% for individual features [15] and 27.5% for a cumulative score threshold [10]—is largely explained by methodological differences rather than by genuine disagreement over the criteria themselves.

Four sources of divergence are relevant. First, the unit of analysis differs: a composite definition classifying a uterus as positive when any single direct feature is present will necessarily yield higher sensitivity and lower specificity than a definition requiring a specific named feature or a cumulative score above a threshold. Second, operator experience varies substantially; single-expert validation in a specialized gynecological ultrasound unit [11] is not comparable with multi-operator or general-practice cohorts, and reproducibility across operators is modest [16]. Third, the composition of the study populations differs: histological prevalence, the proportion of uteri containing leiomyomas—which degrade sonographic detection [6]—and the relative frequency of diffuse versus focal disease all influence both sensitivity and predictive values. Fourth, the reference standard is not uniform because the histological threshold for adenomyosis is not standardized across laboratories, a consideration that applies equally to the present series (Section 2.4).

Taken together, these considerations indicate a consistent underlying pattern—MUSA features are more specific than sensitive—and suggest that published sensitivity estimates should be interpreted in light of the feature definition, the operator, and the cohort’s case mix from which they were derived, rather than treated as a single transferable value.

### 4.8. Clinical and Reproductive Implications

The identification of adenomyosis in 43.7% of hysterectomies underscores that this condition is common in the surgical population and that its clinical relevance extends beyond the operating room. For premenopausal women with fertility intentions, the finding of adenomyosis warrants specific counseling about its adverse effects on fertility and pregnancy outcomes. Moawad et al. reported pooled pregnancy rates of 35–61% after uterus-preserving surgical approaches, with combined surgical–medical therapy yielding superior outcomes [4]. Harada et al. documented substantially elevated obstetric risks in pregnancies affected by adenomyosis, including preterm birth, placental abruption, fetal growth restriction, and preeclampsia [17]. National utilization data from the United States show that hysterectomy remained the predominant inpatient procedure for uterine fibroids and adenomyosis between 2011 and 2020, with uterine artery embolization markedly underutilized [18]. This reliance on surgery illustrates why preoperative recognition matters: earlier identification would allow patients to be counseled on uterus-preserving alternatives, particularly the levonorgestrel-releasing intrauterine device (LNG-IUD), which remains effective even in women at high risk of device expulsion when fixed hysteroscopically [19]. We emphasize, however, that the present study was not designed to test whether structured sonographic assessment improves the detection of adenomyosis; these considerations therefore identify a question for prospective evaluation rather than establish a proven benefit.

### 4.9. Strengths and Limitations

The strengths of this study include a large, consecutive surgical series spanning six years, histopathological confirmation in all cases, a multi-surgeon real-world design, and a systematic comparison with the contemporary international literature.

Two aspects of the histopathological reference standard merit particular emphasis because each may shift the measured prevalence. First, no uniform quantitative depth threshold was mandated for the diagnosis (Section 2.4), and the threshold applied is known to influence prevalence substantially: more permissive criteria—for example, accepting glands and stroma immediately beneath the junctional zone—increase the recorded prevalence, whereas stricter criteria, such as involvement of more than one third of the myometrial wall, reduce it. Series applying different definitions have reported prevalences ranging from below 20% to above 60% [7,8]. Reporting was performed by a single attending pathologist working with the hospital’s resident team over the six-year study period. Without centralized re-review, some variation in the implicit threshold applied—across the different residents involved over time, and potentially by the same observers at different points in the study period—cannot be excluded, and its net direction cannot be determined from the available records. Such misclassification would be expected to be non-differential with respect to the preoperative referral diagnosis and would therefore tend to bias between-group comparisons toward the null rather than generate spurious associations. Second, myometrial sampling was not protocolized, and the number of blocks examined is a recognized determinant of detection: because adenomyosis may be focal, limited sampling will fail to identify disease that more extensive sampling would reveal. This second source of error is directional and can only lead to underestimation, so the true prevalence in this cohort is, if anything, likely to exceed the 43.7% observed.

Further limitations apply. The absence of detailed clinical data—bleeding volume, pain scores, parity, body mass index, and hormonal therapy history—precluded multivariable analysis, so the reported associations, most notably the association with endometrial hyperplasia, remain univariate and require confirmation in adjusted cohorts. Incidental status was classified based on the referral diagnosis rather than on a structured re-evaluation of the original ultrasound reports against the MUSA 2022 criteria, so the study cannot quantify imaging sensitivity. Finally, this is a single-center series, and the case mix of a tertiary referral hospital may not be representative of general gynecological practice. Future prospective studies with standardized pathological sampling, explicit diagnostic thresholds, structured MUSA imaging review, multivariable modeling, and comprehensive clinical data are needed.

## 5. Conclusions

This six-year retrospective cohort study of 430 abdominal hysterectomies performed for benign uterine indications at a tertiary center in Romania found a histopathological prevalence of adenomyosis of 43.7% (95% CI 39.1–48.4%), consistent with contemporary international series. Adenomyosis occurred across all major referral diagnosis categories, and no single preoperative indication predicted its presence. The only significant histopathological association was with endometrial hyperplasia (OR 2.30; adjusted *p* = 0.0014). This unadjusted association suggests, without establishing, a shared estrogen-dependent mechanism.

The most clinically relevant observation is that 98.9% of adenomyosis cases were incidental, with no mention of adenomyosis in the preoperative referral diagnosis, indicating that the condition is rarely suspected before hysterectomy. Because our design cannot directly measure ultrasound performance, this finding reflects low documented clinical suspicion rather than demonstrated imaging failure. A plausible contributor—supported by the literature but not demonstrated here—is that MUSA features are often not documented when a more obvious pathology, typically a uterine fibroid, already explains the patient’s symptoms, meaning that coexisting adenomyosis is neither actively sought nor reported.

These observations provide a rationale for prospectively evaluating the structured application of the revised 2022 MUSA criteria in women undergoing assessment for benign uterine pathology, including those with an obvious concomitant lesion. Whether structured reporting, supported by operator training, would reduce the proportion of adenomyosis recognized only after surgery remains to be tested and cannot be concluded from the present data. Prospective studies correlating structured MUSA reporting with histopathology and applying multivariable analysis to co-findings such as endometrial hyperplasia are required to confirm these observations.

## Figures and Tables

**Table 1:** Demographic and procedural characteristics by adenomyosis status.

Characteristic	Total (*N* = 430)	Adenomyosis (*n* = 188)	No Adenomyosis (*n* = 242)	*p*-Value
Age (years)—mean ± SD	49.7 ± 8.6	50.0 ± 7.6	49.4 ± 9.4	0.459 ^a^
Age—median (IQR)	48 (44–53)	49 (46–51)	47 (43–53)	
Age—range	24–77	32–77	24–77	
Age group—*n* (%)				
<30 years	2 (0.5%)	0 (0.0%)	2 (0.8%)	
30–39 years	37 (8.6%)	12 (6.4%)	25 (10.3%)	
40–49 years	229 (53.3%)	107 (56.9%)	122 (50.4%)	
50–59 years	88 (20.5%)	41 (21.8%)	47 (19.4%)	
60–69 years	52 (12.1%)	21 (11.2%)	31 (12.8%)	
≥70 years	10 (2.3%)	3 (1.6%)	7 (2.9%)	
Surgical procedure—*n* (%)				
M02904—Total hyst. + bilateral adnexectomy	280 (65.1%)			
M02901—Total hysterectomy	100 (23.3%)			
M02902—Total hyst. + unilateral adnexectomy	50 (11.6%)			
Adenomyosis prevalence (95% CI)	43.7% (39.1–48.4%)	—	—	—
Incidental adenomyosis (95% CI)	186/188—98.9% (96.2–99.7%)	—	—	—

^a^ Primary analysis: Mann–Whitney U test (U = 23,474, *p* = 0.112); confirmatory sensitivity analysis: Student’s *t*-test (t = 0.741, *p* = 0.459). Non-significant by both methods. SD, standard deviation; IQR, interquartile range; CI, Wilson score 95% confidence interval. Age was missing for 12 cases (2.8%).

**Table 2:** Preoperative referral diagnoses by adenomyosis status.

Referral Diagnosis	Total (*N* = 430)	Adenomyosis (*n* = 188)	No Adenomyosis (*n* = 242)	OR (95% CI)	*p*-Value
Uterine fibroid/fibromatosis	283 (65.8%)	127 (67.6%)	156 (64.5%)	1.15 (0.77–1.72)	0.570 NS
Abnormal uterine bleeding ^a^	229 (53.3%)	104 (55.3%)	125 (51.7%)	1.16 (0.79–1.70)	0.510 NS
Genital prolapse	40 (9.3%)	18 (9.6%)	22 (9.1%)	1.06 (0.55–2.04)	0.997 NS
Ovarian cyst	25 (5.8%)	15 (8.0%)	10 (4.1%)	2.01 (0.88–4.59)	0.138 NS
Cervical dysplasia/cervicitis	33 (7.7%)	16 (8.5%)	17 (7.0%)	1.23 (0.60–2.51)	0.695 NS
Pelvic/abdominal tumor	6 (1.4%)	3 (1.6%)	3 (1.2%)	1.29 (0.26–6.47)	NS
Postmenopausal bleeding	6 (1.4%)	3 (1.6%)	3 (1.2%)	1.29 (0.26–6.47)	NS
Adenomyosis (preoperative)	2 (0.5%)	2 (1.1%)	0 (0.0%)	—	—

^a^ Abnormal uterine bleeding includes metrorrhagia and/or uterine hemorrhage. Categories are not mutually exclusive. OR, odds ratio; CI, confidence interval; NS, not statistically significant (*p* ≥ 0.05).

**Table 3:** Histopathological co-findings by adenomyosis status.

Histopathological Finding	Adenomyosis (*n* = 188)	No Adenomyosis (*n* = 242)	OR (95% CI)	χ^2^	*p*-Value	Adjusted *p*-Value ^a^
Leiomyoma	142 (75.5%)	167 (69.0%)	1.39 (0.90–2.13)	1.916	0.166 NS	0.581
Endometrial hyperplasia	67 (35.6%)	47 (19.4%)	2.30 (1.48–3.55)	13.461	0.0002 **	0.0014 **
—Simple without atypia	43 (22.9%)	—	—	—	—	—
—With atypia	2 (1.1%)	—	—	—	—	—
—Unspecified type	22 (11.7%)	—	—	—	—	—
Ovarian cysts/pathology	74 (39.4%)	87 (36.0%)	1.16 (0.78–1.71)	0.390	0.532 NS	0.858
Endometrial polyp	13 (6.9%)	21 (8.7%)	0.78 (0.38–1.61)	0.242	0.623 NS	0.858
Endometritis	12 (6.4%)	13 (5.4%)	1.20 (0.53–2.70)	0.056	0.813 NS	0.858
Endometriosis	7 (3.7%)	11 (4.5%)	0.81 (0.31–2.14)	0.032	0.858 NS	0.858
Malignancy (any)	11 (5.9%)	19 (7.9%)	0.73 (0.34–1.57)	0.380	0.537 NS	0.858
—Carcinoma	9 (4.8%)	—	—	—	—	—
—Sarcoma/leiomyosarcoma	2 (1.1%)	—	—	—	—	—

OR, odds ratio; CI, confidence interval; χ^2^, chi-square statistic with Yates continuity correction; NS, not statistically significant (*p* ≥ 0.05); ** *p* < 0.01. Hyperplasia subtypes were reported only for the adenomyosis group. Malignancy subtypes refer only to adenomyosis cases. ^a^ Benjamini–Hochberg false-discovery-rate-adjusted *p*-values across the seven primary co-finding comparisons; Bonferroni- and Holm-adjusted values were identical for endometrial hyperplasia (both 0.0014).

**Table 4:** Histopathological co-findings: present study versus the literature.

Finding	Adenomyosis (*n* = 188)	No Adenomyosis (*n* = 242)	OR (95% CI)	*p*-Value	Literature Range
Leiomyoma	142 (75.5%)	167 (69.0%)	1.39 (0.90–2.13)	0.166 NS	~47% (submucous); 15–57% co-occurrence range
Endometrial hyperplasia	67 (35.6%)	47 (19.4%)	2.30 (1.48–3.55)	0.0002 **	NR in comparable literature—novel finding
Ovarian cysts/pathology	74 (39.4%)	87 (36.0%)	1.16 (0.78–1.71)	0.532 NS	NR
Endometrial polyp	13 (6.9%)	21 (8.7%)	0.78 (0.38–1.61)	0.623 NS	Variable
Endometritis	12 (6.4%)	13 (5.4%)	1.20 (0.53–2.70)	0.813 NS	NR
Endometriosis	7 (3.7%)	11 (4.5%)	0.81 (0.31–2.14)	0.858 NS	~13.5% [6]
Malignancy	11 (5.9%)	19 (7.9%)	0.73 (0.34–1.57)	0.537 NS	NR

OR, odds ratio; CI, confidence interval; NS, not statistically significant; ** *p* < 0.01; NR, not reported.

**Table 5:** Referral-based preoperative detection of adenomyosis in the present study and reported diagnostic performance of imaging modalities in the literature.

Modality	Present Study (Referral-Based)	Maghsoudlou et al. [11]	Lonky et al. [6]	Reported Performance (Literature)
2D Transvaginal Ultrasound	Standard preop; adenomyosis rarely identified	Composite direct: Sn 100%, Sp 35.7%	Sn 47.4%, Sp 50.0% (with fibroids)	~78% Sn without fibroids; degrades with fibroids
MRI	Selected cases only	Not routinely available for all cases	Preferred over US when fibroids present	~80% Sn and Sp; superior to US with fibroids
MUSA 2022 criteria	Not systematically applied	Best marker: subendometrial lines/buds AUC 73.1%	Not applied	Myometrial cysts: Sp 85.7%, Sn 25%
Histopathology (gold standard)	All 430 cases post-hysterectomy	All 26 cases	All 370 cases	Gold standard by definition
Preoperative adenomyosis detection rate	2/188 (1.1%)	N/A	Not reported	Historically very low; major unmet diagnostic need

TVUS, transvaginal ultrasound; MRI, magnetic resonance imaging; MUSA, Morphological Uterus Sonographic Assessment; Sn, sensitivity; Sp, specificity; AUC, area under the curve; N/A, not applicable.

## Data Availability

The data presented in this study are available on reasonable request from the corresponding author. The data are not publicly available because they contain information that could compromise the privacy of the patients included in the study.

## Abbreviations

The following abbreviations are used in this manuscript:

AUB	Abnormal uterine bleeding
CI	Confidence interval
FDR	False discovery rate
IQR	Interquartile range
LNG-IUD	Levonorgestrel-releasing intrauterine device
MRI	Magnetic resonance imaging
MUSA	Morphological Uterus Sonographic Assessment
OR	Odds ratio
SD	Standard deviation
TVUS	Transvaginal ultrasound
